# Adaptation of Mouse Skeletal Muscle to Long-Term Microgravity in the MDS Mission

**DOI:** 10.1371/journal.pone.0033232

**Published:** 2012-03-28

**Authors:** Dorianna Sandonà, Jean-Francois Desaphy, Giulia M. Camerino, Elisa Bianchini, Stefano Ciciliot, Daniela Danieli-Betto, Gabriella Dobrowolny, Sandra Furlan, Elena Germinario, Katsumasa Goto, Martina Gutsmann, Fuminori Kawano, Naoya Nakai, Takashi Ohira, Yoshitaka Ohno, Anne Picard, Michele Salanova, Gudrun Schiffl, Dieter Blottner, Antonio Musarò, Yoshinobu Ohira, Romeo Betto, Diana Conte, Stefano Schiaffino

**Affiliations:** 1 Department of Biomedical Sciences, University of Padova, Padova, Italy; 2 Section of Pharmacology, Department of Pharmacobiology, University of Bari, Italy; 3 Department of Human Anatomy and Physiology, University of Padova, Italy; 4 DAHFMO-Unit of Histology and Medical Embryology, Sapienza University, IIM, Rome, Italy; 5 National Research Council, Institute of Neuroscience, Padova, Italy; 6 Department of Physiology, Graduate School of Health Sciences, Toyohashi Sozo University, Toyohashi-shi, Aichi, Japan; 7 Charité-Universitätsmedizin Berlin, Vegetative Anatomy and Zentrum für Weltraummedizin Berlin, Berlin, Germany; 8 Graduate School of Medicine and Frontier Biosciences, Osaka University, Japan; 9 Venetian Institute of Molecular Medicine, Padova, Italy; Pennington Biomedical Research Center, United States of America

## Abstract

The effect of microgravity on skeletal muscles has so far been examined in rat and mice only after short-term (5–20 day) spaceflights. The mice drawer system (MDS) program, sponsored by Italian Space Agency, for the first time aimed to investigate the consequences of long-term (91 days) exposure to microgravity in mice within the International Space Station. Muscle atrophy was present indistinctly in all fiber types of the slow-twitch soleus muscle, but was only slightly greater than that observed after 20 days of spaceflight. Myosin heavy chain analysis indicated a concomitant slow-to-fast transition of soleus. In addition, spaceflight induced translocation of sarcolemmal nitric oxide synthase-1 (NOS1) into the cytosol in soleus but not in the fast-twitch extensor digitorum longus (EDL) muscle. Most of the sarcolemmal ion channel subunits were up-regulated, more in soleus than EDL, whereas Ca^2+^-activated K^+^ channels were down-regulated, consistent with the phenotype transition. Gene expression of the atrophy-related ubiquitin-ligases was up-regulated in both spaceflown soleus and EDL muscles, whereas autophagy genes were in the control range. Muscle-specific IGF-1 and interleukin-6 were down-regulated in soleus but up-regulated in EDL. Also, various stress-related genes were up-regulated in spaceflown EDL, not in soleus. Altogether, these results suggest that EDL muscle may resist to microgravity-induced atrophy by activating compensatory and protective pathways. Our study shows the extended sensitivity of antigravity soleus muscle after prolonged exposition to microgravity, suggests possible mechanisms accounting for the resistance of EDL, and individuates some molecular targets for the development of countermeasures.

## Introduction

Although adult skeletal muscles are well differentiated into slow- and fast-twitch fibers, they are still able to adapt their phenotype in response to modified functional requests by expressing specific forms or levels of proteins contributing to excitability, E-C coupling, contraction, calcium handling, and energy metabolism [1]. Muscle mass is also critically dependent on chronic functional demand. During spaceflight, the absence of gravity is known to affect mainly the postural, “antigravity” muscles and, to a lesser extent, muscles involved in fast movements [2]–[6]. Reduction of muscle mass (atrophy) and alteration of muscle function during spaceflight cause serious medical problems for astronauts upon return to Earth. Indeed, reduced muscle strength and endurance capacity and enhanced muscle fragility may impair postural maintenance and locomotion activity. Also during a long-duration space mission, the alteration of muscle performance may reduce the ability of astronauts to perform specific tasks. There is therefore a critical need for understanding the molecular mechanisms responsible for muscle impairment to identify possible targets for countermeasures.

Previous studies have shown adverse effects on muscle in spaceflown rats [5], [7] and mice [3]. These changes are qualitatively similar to those observed in humans [8]–[10], including atrophy and partial shift of muscle fibers toward a faster, more glycolytic phenotype [11]. All these studies were performed in spacecraft missions not exceeding 5–20 days (American Shuttle and Russian Cosmos). The results of short-term missions showed that the postural soleus muscle undergoes a rapid and large mass reduction of about 30–40% compared to on ground controls. Moreover, exposure to microgravity produces a substantial shift of soleus muscle toward a faster phenotype, with a significant decrease of type 1 and 2A fibers and increase of 2X and 2B fibers, with changes in corresponding myosin heavy chain (MyHC) isoforms [2], [3], [5], [12]. These morphological and phenotype transformations are associated to a large rearrangement of the expression of various genes, in particular those related to growth/atrophy and cell stress [13], [14]. To complete our understanding of the mechanisms of muscle atrophy and impairment, it is critical to verify the dependence of these effects on the duration of microgravity exposure. The study of long-term missions is especially relevant in view of future missions directed to the Moon or Mars.

With the MDS payload designed by Alcatel-Alenia Space and the Italian Space Agency (ASI), we had the opportunity to accommodate mice aboard the International Space Station (ISS) for 91 days, which represents the longest space journey of rodents up to now. MDS mission was aimed at investigating the responses of various tissues to the prolonged exposure to microgravity. Moreover, the possible protective action of pleiotrophin (PTN) over-expression in microgravity-induced osteoporosis was investigated by utilizing PTN-over-expressing mice. PTN is a diffusible factor usually liberated from osteoblasts and its over-expression is expected to stimulate the osteoblasts and attenuate osteoporosis development [15], [16].

An international muscle team from Italy, Germany and Japan, was appointed by ASI to investigate the effects of long-term microgravity on skeletal muscle. We performed a comprehensive study of the adaptation of mouse hindlimb skeletal muscles to long-term exposure to microgravity. Muscles were removed from mice soon (about 3 hours) after return to Earth aboard the shuttle Atlantis and immediately frozen for subsequent morphologic and gene expression analyses.

The study demonstrates the extended sensitivity to microgravity of antigravity soleus muscle after prolonged exposure to spaceflight, while evidencing possible mechanisms of resistance to microgravity-induced atrophy of the fast-twitch extensor digitorum longus (EDL) muscle. Moreover, the results also indicate that IGF-1, interleukin-6, and NOS1 might represent molecular targets for the development of countermeasures.

## Materials and Methods

### Animals

Wild type male C57BL/10J mice were used for this study. In all phases of the experiment (pre-flight, during the flight and post-flight) handling of animals was in accordance with the principles expressed in the “Guide for the care and the use of laboratory animals” (Office of Science and Health Reports of the USA National Institute of Health, Bethesda, USA). The approval of the MDS experiment was requested and obtained by the American Institutional Animal Care and Use Committee (IACUC protocol n°FLT-09-070 - KSC) as well as by the Ethics Committee of the Animal Facility of the National Institute for Cancer Research (Genoa, Italy) and by the Public Veterinary Health Department of the Italian Ministry of Health (Ministero del Lavoro, della Salute e delle Politiche Sociali prot. n°4347-09/03/2009-DGSA.P.).

The authors of this article were not directly involved in/responsible for designing and/or executing the animal maintenance part of the experiment. Instead they were allowed access to the mice at the end of the flight mission and of the ground control experiments and participated in the specific tissue collection. Additional information about the MDS hardware adopted for housing the animal in space, the animal behavior during the flight and the efforts made to reduce mice pain and suffering during the whole experiment are reported by Cancedda et al. in the companion article “The Mice Drawer System (MDS) Experiment and the Space Endurance Record-Breaking Mice”.

### MDS spaceflight mission

Three groups of wild-type mice, each composed of three animals, were originally planned for this study: three mice housed on ground for 91 days in normal laboratory cages (LAB); three mice housed on ground for 91 days in MDS payload (Ground); three spaceflight mice housed in MDS payload for 91 days on board ISS (Flight). Moreover, three similar groups of PTN-overexpressing mice were part of the study. However, as PTN effects on skeletal muscle are not yet established, the PTN-overexpressing mice were not included in the present study. The MDS payload developed by Alenia-Space [15] and the MDS mission characteristics are described in the leading article of this series. Mice were transported to the ISS on board the Shuttle Discovery (NASA mission STS-128) and back to the Earth on board the Shuttle Atlantis (mission STS-129; August–November, 2009). The on-ground experiment was carried out at the Animal Housing Facility of the Advanced Biotechnology Center in Genova. Two wild type mice died on board the ISS for unpredictable episodes (see leading article) so that the analysis was focused on one spaceflown mouse and on the corresponding ground controls. The EDL and soleus muscles of mice killed by carbon dioxide inhalation were excised bilaterally at the Life Sciences Support Facility of Kennedy Space Center within 3 hours after the Shuttle landing.

### Morphological analysis

Muscles were frozen in liquid nitrogen in a slightly stretched position. Serial cross sections (8-µm thick) were cut in a cryostat microtome set at −24±2°C (Slee Pearson, UK). For the histological analysis, hematoxylin-eosin staining was performed on muscle sections to examine the general morphology. To measure the cross-sectional area (CSA) of individual fibers, muscle cryostat section were stained for laminin, a major component of the basal lamina. Digital photographs were taken of each muscle section and the CSA was automatically measured as the internal laminin-unstained area by the ImageJ 1.45 g (NIH, freeware imaging software). A minimum of 300 fibers per muscle were measured.

### Analysis of myonuclei and satellite cells

To count muscle fiber nuclei the cryosections were fixed with 4% paraformaldehyde for 15 min. Blocking was performed using mouse IgG blocking reagent (M.O.M. Vectorlabs) and 10% donkey serum (Sigma, USA) in 0.1 M phosphate-buffered saline (PBS) containing 0.1% triton X-100 for 1 hr. Muscle fibers were stained for laminin by using a specific rabbit polyclonal antibody (Sigma, USA), diluted 1∶100 with 0.1 M PBS containing M.O.M. protein concentrate stock solution and 0.1% triton X-100 over night at 4°C. The secondary antibody, Alexa fluor 488 donkey anti-rabbit IgG antibody (Invitrogen, USA), diluted 1∶200 with 0.1 M PBS containing M.O.M. protein concentrate stock solution and 0.1% triton X-100, was added for 4 h at room temperature in the dark. Finally, fluorescent-labeled muscle sections were mounted on a slide glass by using VECTASHIELD mounting medium with 4′,6-diamidino-2-phenylindole (DAPI, Vector Laboratories, USA) to stain nuclei. Myonuclear number per fiber cross-section was identified by counting nuclei, which were located inside of laminin. Approximately 100 fibers were analyzed in two regions of each muscle sample. A fluorescence microscope (BX50, Olympus, Japan) was used to visualize the stained images.

Immunohistochemical staining for Pax7 was used to identify satellite cells. Staining was performed in a different muscle section by using M.O.M. basic kit (Vector Laboratories, USA) after antigen retrieval. Muscle sections covered with 10 mM sodium citrate buffer were heated once using a microwave and then probed with the primary antibodies (anti-Pax7, R&D Systems, Inc., USA, and anti-laminin, Sigma-Aldrich, USA) after fixation and blocking mouse IgG, as specified above. Alexa Fluor 488 donkey anti-mouse IgG antibody and Alexa Fluor 594 donkey anti-rabbit IgG antibody (Invitrogen, USA) were used as the secondary antibodies. Fluorescent-labeled muscle sections were then mounted on a slide glass by using VECTASHIELD mounting medium with DAPI (Vector Laboratories, USA) to stain nuclei and the fluorescent images were acquired into a computer. Number of Pax7-positive cells, located inside of the laminin layer, per muscle cross-section was counted.

### Nitric oxide synthase-1 (NOS1) immunofluorescence analysis

Cryosections of cross-sectioned skeletal muscle mounted on super-frosted slides (Thomas) were fixed in 4% paraformaldehyde for 10 min at 4°C and processed for NOS1 immunofluorescence histochemical analysis. Specimens were preincubated with the mouse Ig blocking reagent (M.O.M., Vectorlabs) in order to block endogenous mouse IgG background. We used anti-NOS1 monoclonal (raised against the N-terminal amino acid 2–300, Santa Cruz) and/or polyclonal (Sc 648, Santa Cruz) antibodies together with anti-fast MyHC (Clone My32, Santa Cruz) in double staining experiments. Primary antibodies were visualized by using Alexa 488-conjugated and/or Alexa 555-conjugated affinity-purified goat anti-mouse and anti-rabbit secondary antibodies (Invitrogen). Specimens were analyzed by routine epifluorescence (Zeiss Axioplan) or confocal microscopy (Leica Microsystems, Mannheim, Germany). For NOS1 confocal microscopy analysis, immunofluorescence images were obtained by using a three channel confocal laser scanning microscope (Leica TCS SP-2, Leica Microsystems, Bensheim, Germany), as previously described for NOS1-3 isoforms [17]. The relative fluorescence intensity of NOS1 immunostained skeletal muscle structures was measured [18]. Briefly, the area pixel intensity of a selected region of interest (ROI) of type 1 or type 2 fiber was measured in digitized confocal image scans and expressed as arbitrary units (a. u.), in the range of 0 to 255 a. u.. At least twenty (n = 20) type 1 and/or type 2 myofibers were selected from each cryosection/muscle sample. Changes of intensity referable to NOS1 were calculated as ratio a.u. of sarcolemmal vs. cytoplasmic immunolocalization. All digitized images were analyzed using the Leica confocal software. For statistical analyses, we used the SigmaPlot Version 9.0 software. The results are given as mean (± SEM). Significance of differences of data was analyzed with Student's *t*-test. Differences were regarded to be statistically significant at p<0.05.

### Immunofluorescence fiber typing

Fiber typing was determined by immunofluorescence using combinations of the following monoclonal antibodies: BA-D5 that recognizes type 1 MyHC isoform; SC-71 for type 2A MyHC isoform; BF-F3, for type 2B MyHC isoform [19]. To detect the primary antibodies the following secondary antibodies were used: DyLight405 labeled goat anti mouse IgG, Fcγ 2b subclass specific (115-475-207), to specifically detect BA-D5, DyLight488 labeled goat anti mouse IgG, Fcγ 1 subclass specific (115-485-205), for SC71, and DyLight549 labeled goat anti mouse IgM (115-505-075), used to specifically detect BF-F3. Secondary antibodies were purchased from Jackson Immunoresearch, anti-HA, 16B12, from Covance, USA, and anti-myc, 9E10, from Roche. Pictures were collected with an epifluorescence Leica DM5000 B equipped with a Leica DFC 300 FX camera. Single-color images were merged to obtain a whole muscle reconstruction with Adobe Photoshop CS2 (Adobe Systems Inc.).

### SDS-PAGE of muscle extracts

Analysis of MyHC isoforms was performed as previously described [20]. Shortly, small muscle fragments from control and MDS muscles were weighed, ground with a ceramic pestle in liquid nitrogen, and extracted at 2 mg/ml in SDS-PAGE sample buffer (62.5 mM Tris, pH 6.8, 2.3% SDS, 5% 2-mercaptoethanol, 10% glycerol). Forty µg of muscle sample was run on 8% SDS-PAGE slab gels in SDS. MyHC protein bands from whole muscles were revealed by Coomassie brilliant blue staining. MyHC isoform percentage composition was determined by densitometry of gels by using a Bio-Rad Imaging Densitometer (GS-670).

### RNA extraction and reverse transcription

Total RNA was extracted by using the Qiagen RNeasy Micro Kit. The extracted RNA was eluted in 14 µl RNase-free water, analyzed by capillary electrophoresis (RNA 6000 Pico LabChip, Agilent Technologies) and stored at −80°C until used. Quantification was performed in a 96-well IQ5 Thermal Cycler (Bio-Rad). Due to the low amount of RNA obtained from the small specimens, amplification was necessary and was carried out by the Ovation Pico kit from NuGEN, according to manufacturer's indication. The cDNA product was then utilized for the quantitative PCR analysis.

### Quantitative PCR

A panel of genes was considered in the study. To quantitate the expression of the genes diverse RT-PCR techniques were utilized, as indicated in the **Table S1** and briefly described below. Transcript levels of Murf-1, atrogin-1, LC3b, cathepsin L, PGC-1a and MRF4 were quantitated by the SYBR Green method. Specific PCR primers (**Table S1**) were from Eurofins MWG Operon. The reaction mix consists of 10 µl of 2× iQ SYBR Green Supermix (Bio-Rad), 0.3 pmol/µl primers, 2 ng of cDNA and DNase/RNase-free water up to 20 µl. The PCR parameters were initial denaturation at 95°C for 30 s followed by 40 cycles of 10 s at 95°C and 30 s at the corresponding annealing temperature (55–59°C) for acquisition of fluorescence signal. A melting curve was generated by the iQ5 software following the end of the final cycle for each sample, by continuous monitoring the SYBR Green fluorescence throughout the temperature ramp from 65°C to 99°C in 0.5 s increments. The mRNA expression of the genes of interest in each experimental condition was normalized to the housekeeping genes (cyclophilin A and β-actin).

Transcript levels of stress-related genes, as well as Pax7 and interleukin-6 genes, were quantitated by the SYBR Green method as follows. Synthesized cDNA was applied to real-time reverse transcription-PCR (Thermal Cycler Dice® Real Time System II MRQ, Takara Bio Inc.) using Takara SYBR *Premix Ex Taq*™ II for mRNA, and analyzed with Takara Thermal Cycler Dice® Real Time System Software Ver. 4.00 according to the manufacturer's instructions. The real-time cycle conditions were 95°C for 30 s followed by 40 cycles at 95°C for 5 s and at 60°C for 30 s for mRNA. Specificity was confirmed by electrophoretic analysis of the reaction products and by inclusion of template- or reverse transcriptase-free controls. To normalize the amount of total RNA present in each reaction, GAPDH cDNA for mRNA was used as an internal standard. The primers were designed by using the Takara Bio Perfect Real Time Support System (Takara Bio Inc.).

Transcript levels of plasma membrane ion channels and PKC isoforms genes were quantitated by the Taq polymerase method. Real time TaqMan MGB experiments were performed using specific primer and probe sequences designed by ourselves or obtained from Applied Biosystems (**Table S1**). To achieve a high level of specificity and to avoid detection of genomic DNA, the probe was designed to span exon-exon junctions for each gene. The PCR was run for 10 min at 95°C for activation enzyme, 45 cycles for 6 s at 95°C for denaturating, and 6 s at 60°C for annealing and extension. Each reaction (25 µl) contained 8 ng of cDNA 1 pmol of each primer, 10 pmol of probe and 12.5 µl of TaqMan Universal PCR Master Mix, No AmpErase UNG. The target gene was tested as internal control, together with the housekeeping gene hypoxanthine guanine phosphoribosyl transferase 1 (HPRT1), as previously described [21]. Triplicate reactions were carried out in parallel for each individual muscle sample, and the results were compared with a gene-specific standard curve. The HPRT1 gene was used for normalization of target genes signal.

Transcript levels of IGF-1 isoforms were quantitated by the Taq polymerase method, utilizing specific probes (**Table S1**) and conditions recommended by Applied Biosystems.

## Results

### Morphometric and immunohistochemical analyses

Morphological analysis showed that prolonged exposure to microgravity did not cause the development of abnormalities or pathological signs in EDL and soleus muscles (**Fig. S1**). The absence of centronucleated fibers and lack of significant expression of embryonic MyHC and of the Nav1.5 sodium channel subtype (data not shown), typically expressed in neonatal or denervated muscles but absent from healthy adult muscles [22], argue against degeneration/regeneration events in both soleus and EDL muscles. Satellite cells have been reported to be reduced in number after hindlimb unloading in adult rat muscles [23]–[25] but were not counted in previous spaceflight studies. We found that after 91 days in space, the mean number of Pax7-positive satellite cells per cross-section of EDL and soleus muscle, was in the control range, i.e. between ∼20 and 40 (data not shown). Also transcript levels of Pax-7 remained unchanged after space flight (see gene expression below), consistent with the knowledge that skeletal muscle adaptation to inactivity is a physiological response not associated to pathological events. In addition, the number of muscle fiber nuclei was reported to decrease in adult rats following hindlimb unloading [23]–[25]. The present study also shows a reduction of the mean number of myonuclei after prolonged exposure to microgravity in soleus muscle (**Fig. S2**).

**Figure 1:**
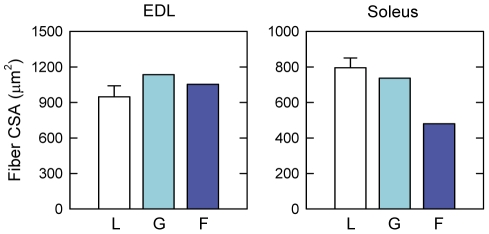
Muscle fiber size of mice flown on board ISS and ground-based controls. Exposure to microgravity for 91 days caused the reduction of the mean cross-sectional area (CSA) of soleus fibers, but not of extensor digitorum longus (EDL) fibers. Laboratory control (L) mice were housed in normal laboratory cages for 91 days (open bars, n = 3, data expressed as means ± SEM). Ground control (G) and spaceflown (F) mice, were housed in MDS (mice drawer system) payload for 91 days on ground (light blue bars; n = 1) and on board the International Space Station (dark blue bars; n = 1), respectively.

To evaluate the microgravity-induced atrophy, the CSA of EDL and soleus muscle fibers was measured on laminin-stained muscles (**Fig. S3**). The data show that EDL muscle fibers do not undergo atrophy in the spaceflown mouse, notwithstanding the long exposure to space microgravity. On the contrary, soleus muscle shows a homogenous reduction of CSA of about 35% (**Fig. 1**). A similar ∼35% reduction was observed when the muscle fiber CSA was normalized to the body weight (**Fig. S4**).

**Figure 2:**
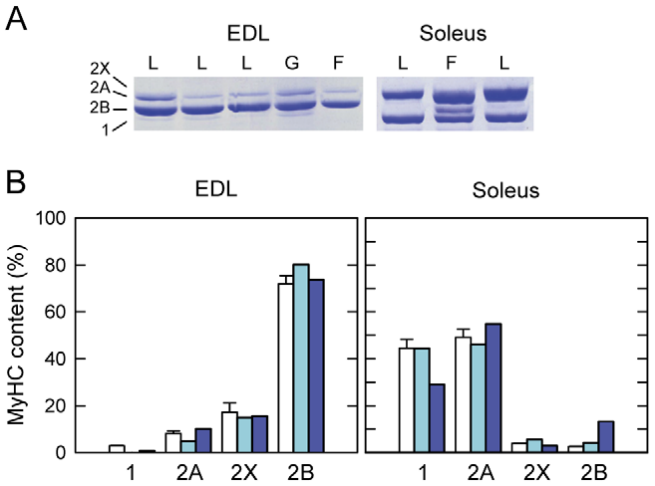
MyHC isoform composition of muscles from mice flown on board ISS and ground-based controls. **A.** Representative SDS-PAGE of myosin heavy chain (MyHC) isoform protein bands from EDL and soleus muscle lysates from ground control mice housed for 91 days in a normal laboratory cage (L) or in the MDS payload (G) and from the spaceflight mouse housed in the MDS payload on board ISS for 91 days (F). **B**. Mean content of MyHC isoform protein bands of laboratory controls housed in laboratory cages (open bars, n = 3, data expressed as means ± SEM), ground control, housed in MDS payload (light blue bars; n = 1), and spaceflight mouse in MDS payload on board ISS for 91 days (blue bars; n = 1).

Muscle disuse is known to cause a slow-to-fast phenotype change, particularly in muscles more frequently utilized, like the antigravity soleus, and less evident in phasic muscles, like the fast-twitch EDL. The expression pattern of MyHC isoforms was measured using SDS-PAGE of solubilized muscle fragments (**Fig. 2A**). The results showed no significant change in MyHC content of the fast-twitch EDL muscle. In contrast, space flown soleus showed a reduction of the slow type 1 MyHC, while the proportion of type 2A and 2B MyHC isoforms increased after spaceflight, suggesting a slow-to-fast transition of soleus muscle (**Fig. 2B**).

**Figure 3:**
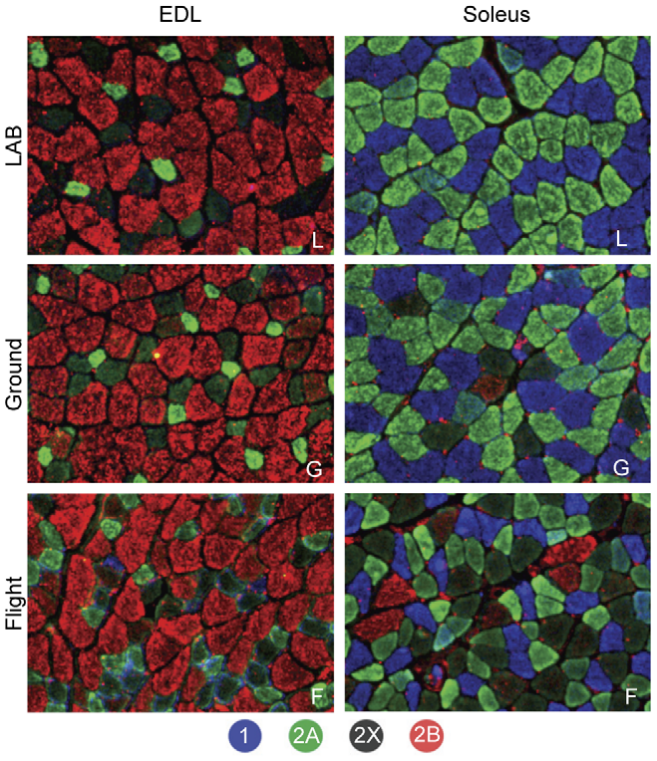
Fiber typing of muscles flown on board ISS for 91 days and ground-based controls. Immunofluorescence fiber type identification by means of monoclonal antibodies specific for the different myosin heavy chain isoforms. The different fiber types were identified as described in Materials and Methods. Note the *de novo* appearance of type 2B fibers. The figure also illustrates the general atrophy of spaceflown soleus muscle fibers, compared to laboratory and ground controls.

**Figure 4:**
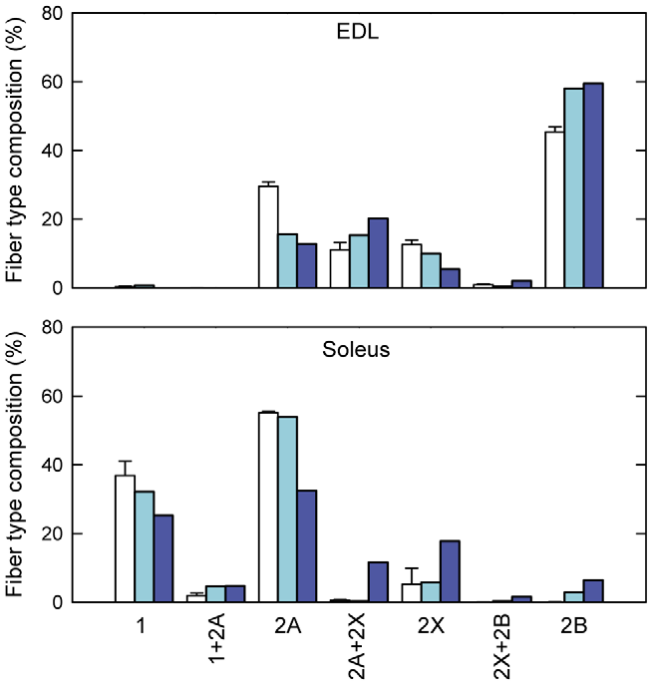
Fiber type composition of muscles from mice flown on board ISS and ground-based controls. Fiber type compositions of EDL and soleus muscles from mice housed in normal laboratory cage (open bars; n = 3, data are expressed as means ± SEM), ground control housed in MDS payload (light blue bars, n = 1), and spaceflight mouse housed in MDS payload (blue bars; n = 1). The prolonged exposure to the microgravity environment caused a slow-to-fast rearrangement of fiber type composition in soleus muscle, but not in EDL.

To determine the fiber type compositions of EDL and soleus muscles, serial muscle cryosections were stained with monoclonal antibodies specific for type 1 (slow), 2A and 2B MyHC subtype, type 2X fibers were identified by the absence of reactivity with the three antibodies (**Fig. 3**). The most striking change was the appearance of type 2B fibers in space flown soleus muscle, as well as the reduction of type 1 and 2A fiber size. Quantitative analysis confirmed the fiber type transformation of soleus muscle, with a reduction of pure type 1 and 2A fibers, and an increase in fibers co-expressing type 2A and 2X, and pure 2X and 2B fibers (**Fig. 4**).

**Figure 5:**
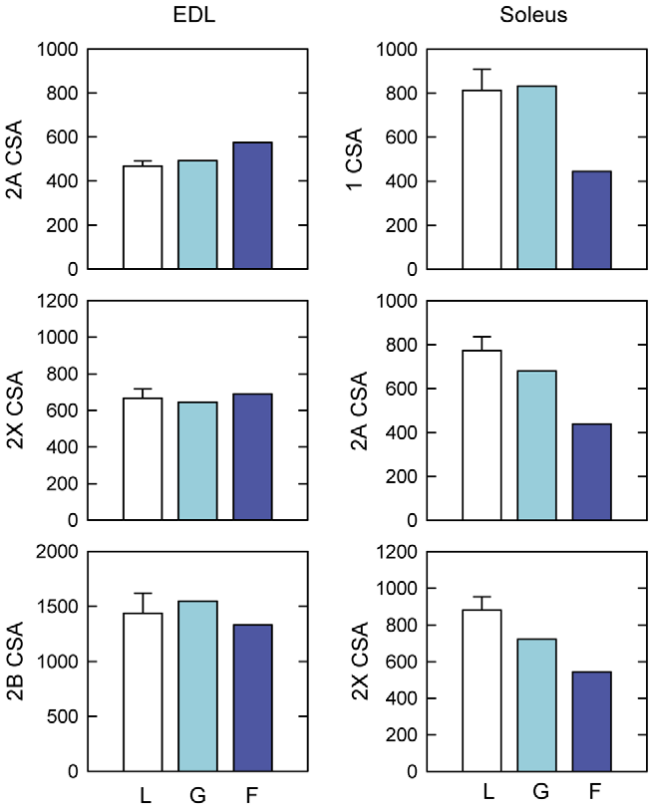
CSA of different fiber types in muscles from mice flown on board ISS and ground-based controls. The CSA was measured in individual fiber types in normal laboratory cage controls (open bars, n = 3, data are expressed as means ± SEM), ground control in MDS payload (light blue bars, n = 1), and spaceflight mouse housed in MDS payload for 91 days on board ISS (blue bars, n = 1). A similar CSA reduction of about 35% was observed in all soleus fibers independently of the fiber type, whereas no changes were evident in EDL fibers.

To verify whether muscle fiber atrophy might differ between the various fiber types, we measured the CSA of fibers identified according to the MyHC isoform they express (**Fig. 5**). The number of pure type 1 fibers in EDL and that of pure type 2B fibers in soleus were too low to be included in the analysis. The CSA of fast fibers, both type 2A, 2X and 2B, remained unchanged in spaceflown EDL compared to on-ground controls (**Fig. 5A**). The analysis of the mean CSA of soleus type 1, 2A, and 2X fibers showed that atrophy developed similarly in all soleus fiber types independently of the MyHC isoform they express (**Fig. 5B**).

**Figure 6:**
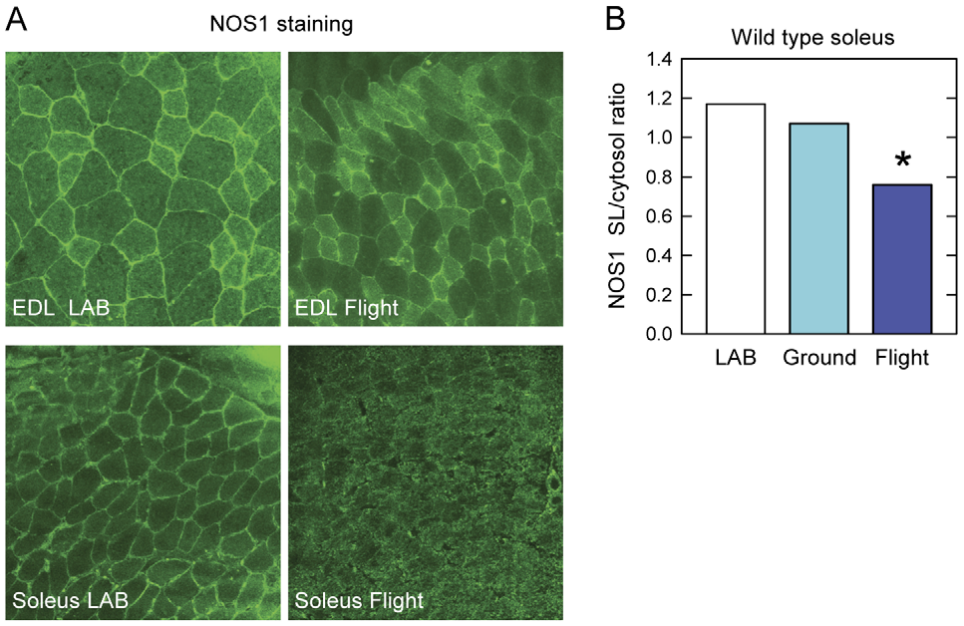
Mislocalization of NOS1 in muscle fibers from mice flown on board ISS. **A.** Confocal immunofluorescence analysis of NOS1 localization in soleus and EDL muscle fibers from ground control mouse housed in a normal laboratory cage (LAB) and of the spaceflight mouse housed in the MDS payload (Flight). NOS1 mislocalization was seen in soleus muscle of the spaceflown mice, but not in EDL. **B**. The sarcolemmal (SL) to cytoplasmic localization of nitric oxide synthase-1 (NOS1) isoform was analyzed in soleus muscle fibers of control mice housed in a normal laboratory cage (LAB) or in the MDS payload (Ground) and in mice flown for 91 days on board ISS (Flight). *: P<0.05 vs. Lab and Ground.

In mice, the nitric oxide synthase NOS1 subtype is usually expressed in both type 1 and type 2 muscle fibers, being almost equally localized in the sarcolemma and in the cytoplasm [26], [27]. To determine whether prolonged exposure to microgravity might affect the distribution of NOS1 in flown muscle fibers, we performed a detailed confocal immunofluorescence analysis. Long term exposure to microgravity determined a substantial change in the ratio of cytosolic to membrane NOS1 staining in soleus muscle but not in EDL (**Fig. 6**). Laser confocal microscope image analysis clearly showed in fact that in soleus muscle the sarcolemmal staining for NOS1 is almost indistinguishable from the cytosolic one, whereas in flown EDL a clear-cut sarcolemmal localization is evident (**Fig. 6A**).

### Gene expression analyses

**Figure 7:**
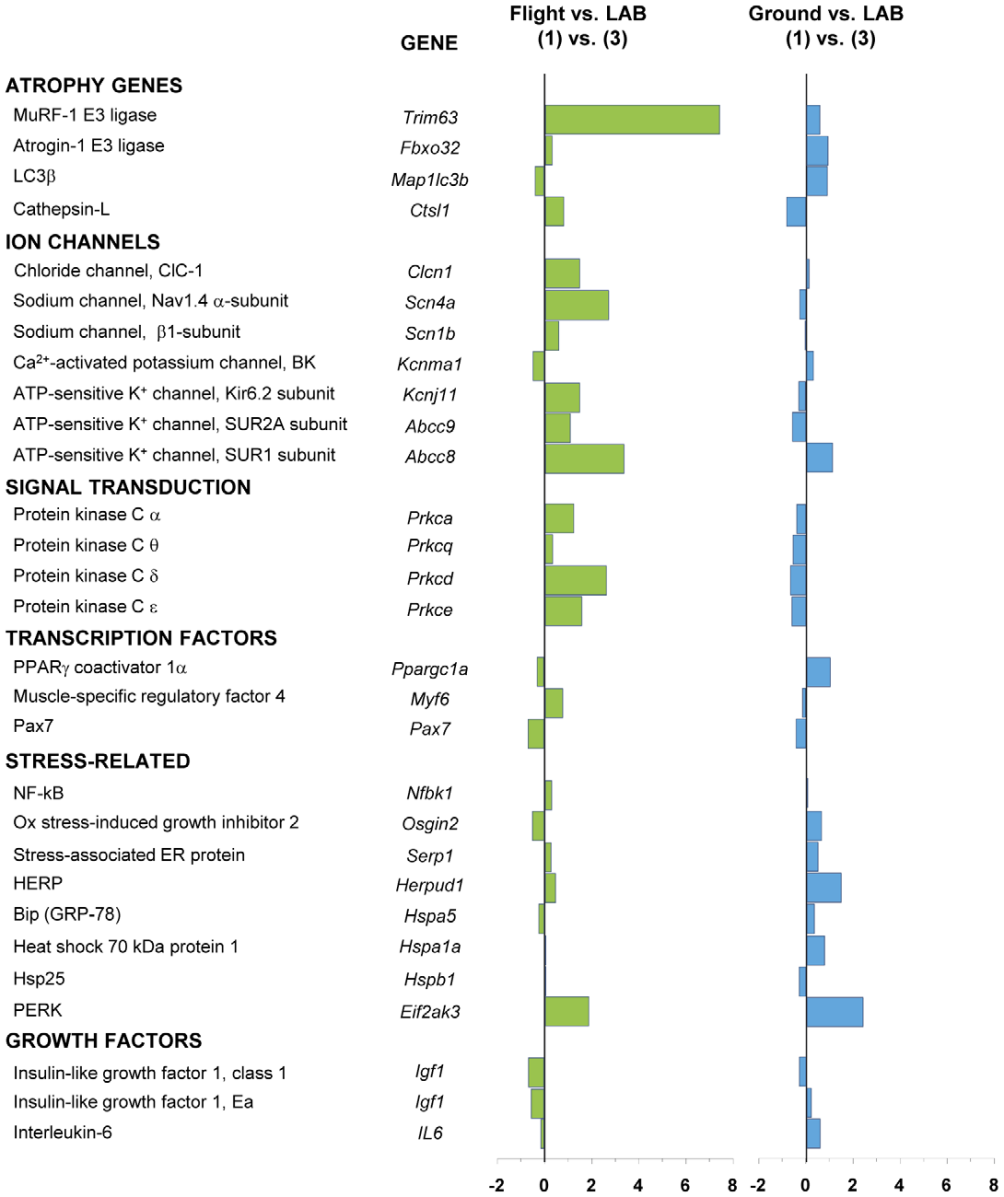
Variations in gene expression in soleus muscle induced by spaceflight. Transcript levels were determined by real-time PCR for selected genes, classified on the basis of the functional role of the protein they encode. The numbers on the abscissa indicate the fold change in gene expression normalized for housekeeping gene. The bars in green show the variation between the spaceflown mouse (Flight) and LAB mice, whereas the bars in blue show the variation between Ground and LAB control mice.

**Figure 8:**
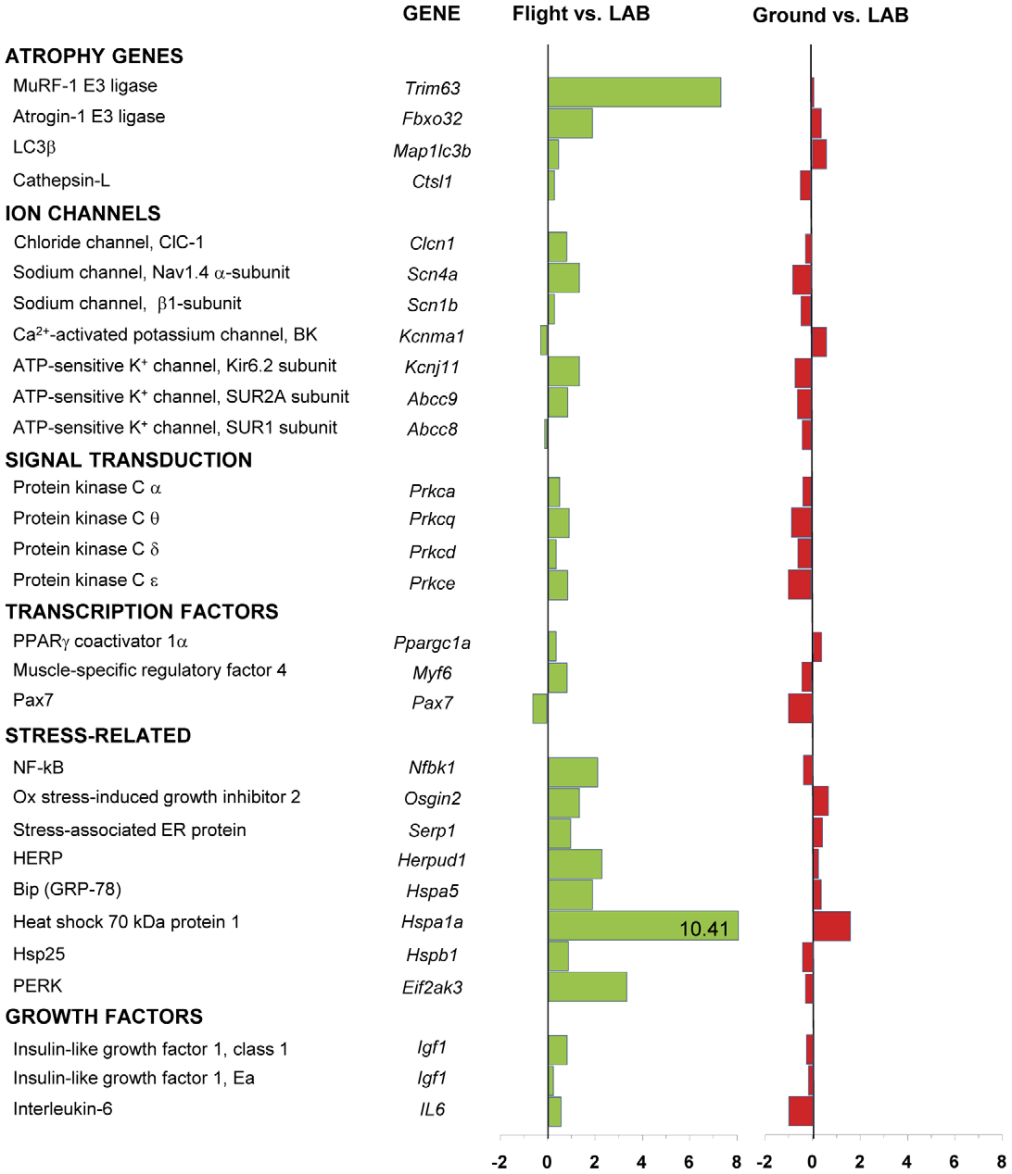
Variations in gene expression in EDL muscle induced by spaceflight. Transcript levels were determined by real-time PCR for selected genes, classified on the basis of the functional role of the protein they encode. The numbers on the abscissa indicate the fold change in gene expression normalized for housekeeping gene. Numbers in the horizontal bars indicate the fold change when out of scale. The bars in green show the variation between the spaceflown mouse (Flight) and LAB mice, whereas the bars in blue show the variation between Ground and LAB control mice.

To determine adaptation changes of gene expression due to prolonged exposure to microgravity, we used RT-PCR to quantify shifts in mRNA levels of a selected panel of genes involved in muscle atrophy and plasticity. Results from RT-PCR are illustrated in **Figure 7** (soleus) and **Figure 8** (EDL), which show the comparison between the spaceflown and LAB mice (column 1) and between Ground and LAB mice (column 2). The comparison between Ground and LAB mice shows that housing in MDS was without major effects on soleus and EDL muscles. One exception is represented by the high level of PERK in Ground soleus compared to LAB, a response that could be due to the more restricted space available in the space housing payload.

We examined the expression of two genes coding for proteins associated with the ubiquitin-proteasome system, MuRF-1 and atrogin-1 (MAFbx), and two genes coding for proteins associated with the autophagy-lysosome system, LC3β and cathepsin-L. Spaceflight dramatically increased the expression of MuRF-1 in both soleus and EDL muscles, while another muscle-specific E3 ubiquitin-ligase, atrogin-1 (MAFbx), was up-regulated only in the EDL muscle. Interestingly, expression of LC3β and cathepsin-L was unmodified both in EDL and soleus. Expression of growth factors, IGF-1 and IL-6, was reduced in soleus muscle, but increased in EDL.

In short term missions, the expression of stress-related heat shock proteins was reduced in rat soleus muscle and unmodified in the fast-twitch plantaris [28]. Quite surprisingly, the expression of various stress-related markers, including heat shock proteins, was dramatically increased in EDL muscle exposed to the microgravity environment for 91 days, whereas no major changes were observed in soleus muscle (**Figs. 7** and **8**).

Disuse atrophy is known to cause down-regulation of PKC α, ε, and θ protein isoforms in both EDL and soleus muscles of hindlimb unloaded rats [29]. **Figures 7** and **8** show that the expressions of α, β, and ε PKC genes were increased in spaceflown soleus, whereas variable and less ample changes were observed in EDL.

Regarding ion channel subunits, spaceflight induced an increased expression of ClC-1, Nav1.4, and ATP-sensitive K^+^ channels and a reduced expression of Ca^2+^-activated K^+^ (BK) channels. All these changes were similar to those observed in hindlimb-unloaded rodents and are in agreement with the slow-to-fast shift of soleus muscle phenotype [30], [31]. Similar, though less ample, effects were found in EDL, suggesting that this fast-twitch muscle may also become faster in actual microgravity.

## Discussion

### Muscle fiber atrophy

Following 91 days of long-term exposure to real microgravity in Space, atrophy was evident in soleus muscle, but was absent in EDL muscle. Soleus muscle atrophy might be probably mainly due to activation of ubiquitin-dependent proteasome activation, since MuRF-1 expression was largely increased while no variation in the expression of autophagy-related genes was found. Quite surprisingly, the expression of both ubiquitin ligases, MuRF-1 and also atrogin-1, was dramatically increased in the non-atrophic EDL muscles, suggesting that this muscle may activate protective or compensatory mechanisms. Interestingly, expression of growth factors, IGF-1 and IL-6, was reduced in soleus muscle and increased in EDL muscle, suggesting that growth pathways may be impaired in soleus but not in EDL muscle. Indeed, the expression of atrophy-related genes is negatively modulated by IGF-1, a growth factor involved in several anabolic pathways of skeletal muscle [32], [33]. Also, the IL-6 gene deletion has been recently shown to blunt muscle hypertrophy [34] and delay recovery of gastrocnemius muscle from hindlimb unloading atrophy, suggesting a protective role of the interleukin in disuse atrophy [35]. This might explain fact that a significant CSA reduction is seen only in the soleus but not in the EDL muscle of spaceflown mice. Thus, the up-regulation of IGF-1 and IL-6 in EDL might be a compensatory mechanism that counteracts muscle atrophy and may be considered as good candidates for the development of countermeasures. Accordingly, expression of the eukaryotic translation initiation factor 2 α kinase (aka PERK) was significantly increased in EDL, suggesting an activation of protein synthesis.

In addition, many stress response genes, including heat shock proteins and ER stress-related proteins, were also up-regulated in the EDL muscles of spaceflown mice, pointing to the activation of protective pathways. Interestingly, while heat shock protein expression was shown to be reduced by hindlimb suspension or 9-day spaceflight in atrophic soleus, but not in the non-atrophic plantaris muscle, heat preconditioning has been shown to prevent partially atrophy and slow-to-fast shift in soleus muscles of hindlimb-unloaded rats [28], [36], [37] and the recovery from atrophy was promoted by application of heat [38]. Beneficial effects of heat stress on increase in protein content of cultured skeletal muscle L6 [39] and C_2_C_12_ cells [40], as well as *in vivo* muscle mass [41], were also reported. It was also recently shown that the ERAD molecule Herp, encoded by Herpud1, whose expression is increased in space flown EDL but not soleus, may delay the degradation of cytosolic proteins at the ubiquitination step [42]. Thus it is likely that up-regulation of these genes during spaceflight may contribute to the protection of EDL muscle.

Our data show that the prolonged exposure of mice to spaceflight caused a proportional atrophy in type 1, 2A, and 2X fibers of soleus muscle whereas atrophy was absent in all fiber types of EDL muscle. Consistently, Harrison et al. [3] showed that exposure of mice to spaceflight for 11 days mice produced large atrophy of a similar extent in type 1 and 2A soleus fibers, whereas no information is available for EDL, as the muscle was not included in the study. By contrast, in the same study the authors showed that gastrocnemius and plantaris underwent less atrophy than soleus, with type 1 and 2A gastrocnemius fibers more atrophic than 2X and 2B, whereas in plantaris type 2B were much more atrophic than 2A and 2X [3]. In on ground mice Hindlimb unloading (HU) was found to induce 42% reduction of soleus muscle mass and 17% in gastrocnemius [43] with a similar atrophy to type 1 (37–59%) and 2A (46–61%) fibers in soleus [44]. Other studies showed that HU causes atrophy of soleus, plantaris, gastrocnemius and tibialis anterior mass without affecting the mass of EDL [45], [46].

In the rat, short exposure to microgravity resulted in substantial atrophy in postural antigravity muscles, however, atrophy appears to be more marked in type 1 than in type 2A fibers of soleus muscle [5], [12], [47], [48], [49]. Contrasting results are reported for fast muscles. A significant reduction of type 2B fibers size was observed in rat EDL after 10 days of spaceflight [50], in all fast fibers after 12.5 days spaceflight [51] and in all but 2X and hybrid 2X/2B fibers after 14 days [52]. By contrast, it has been reported that 9-day spaceflight caused 32% atrophy on rat soleus but not on EDL [53]. Moreover, no atrophy was observed in gastrocnemius and tibialis anterior of rat spaceflown for 14 days [54]. In on ground microgravity experiments, 14-day HU caused atrophy of soleus but not of EDL [55]. Similarly, 14-day HU caused atrophy of soleus but not plantaris [28], gastrocnemius and tibialis anterior [5]. Again, fiber CSA reduction in soleus after 14-day HU was greater in slow- than in fast-twitch fibers [5]. Altogether these data indicate that muscle atrophy associated to both real and simulated microgravity depends more on whole muscle function than on muscle fiber type. The least used ankle extensors, such as EDL, are barely sensitive to unweighting, whereas the antigravity soleus, usually continuously at work, suffers the most dramatic consequences.

It was reported that growth-associated enlargement of soleus muscle fibers was inhibited by 3-month hindlimb unloading after birth in rats [56]. Such phenomena were closely related to the inhibited increase of satellite cell and myonuclear number. In adult rats, hindlimb unloading caused the reduction of myonuclear number in soleus associated with atrophy [23]–[25]. We also observed a trend to the reduction of myonuclei per fiber cross-section after spaceflight. Conversely, no variation of satellite cell number and activation was found in spaceflown mouse soleus muscles, suggesting that the reduction of myonuclei is not compensated by satellite cells-mediated regeneration, and may therefore significantly contribute to muscle fiber atrophy.

In skeletal muscle, expression of the neuronal isoform NOS1 [27], [57] and its muscle-specific splice variant NOSμ [58] shows a marked subcellular partitioning. NOS1 is concentrated at the sub-sarcolemmal region in normal skeletal muscle fibers [27] where it is associated via syntrophin to the dystrophin-glycoprotein (DAG) complex, whereas it is absent from such a membrane location in DAG gene-deleted mutant mice [26]. Mechanical loading vs. unloading has been previously shown to reduce sarcolemmal expression of NOS1 in tail-suspended rats [59], [60] as well as in bedrest [17], supporting a predominant role of NOS as a load sensitive biomarker of skeletal muscle activity/inactivity. In addition, a link between NOS/NO signaling and the Foxo/Akt/mTOR pathway has been shown, suggesting that NO signals might contribute to muscle protection against atrophy [59], even in a fiber-type specific manner [61]. In the present study, we demonstrated for the first time that the NOS1 sarcolemma-to-cytosol ratio is substantially changed in the soleus muscle fibers after the prolonged exposure to spaceflight. This is in agreement with the sarcolemma-to-cytosol NOS1 mislocalization observed in ground-based microgravity models [17], [59], [60]. Though spaceflight-related changes in synthesis/degradation of NOS1 protein might have been occurred, NOS1 mislocalization might be related to microgravity-induced changes in the sarcolemma membrane scaffold associated with the DGC [26].

### Muscle fiber type transition

The short-term exposure to microgravity is known to cause a rapid shift of antigravity soleus muscle toward a faster phenotype, while the already fast-twitch EDL is less responsive [6]. Short-term space missions demonstrated in fact a decrease of type 1 and type 2A fibers in rat soleus muscle and an increase of hybrid fibers [5], [12], [48]. In contrast, short-term spaceflights had no effect on fiber type composition in fast-twitch muscles [4], [62], although a decrease in 2X MyHC isoform has been reported in the EDL muscle of rats flown for 10 days in space [50]. In mice, exposure for 11 days to the microgravity environment was associated with an increase of the percentage of fibers expressing type 2X and 2B MyHC in slow muscles, without significant changes in fast-twitch muscles [3].

The long-term exposure to microgravity on board ISS substantially confirmed the effects on muscle phenotype observed following short-term missions. Analysis of MyHC proportion and of fiber type composition clearly demonstrated the slow-to-fast shift of soleus muscle during spaceflight. The expression of ion channels subunits was also changed in agreement with the phenotype shift [30], [31], indicating that the entire fast-gene program may be activated during spaceflight. Although MyHC expression was not changed in the EDL muscle, except for the *de novo* appearance of pure type 2B fibers, the changes in ion channel expression suggested that the fast gene program was up-regulated also in the EDL muscle. PKC activity is known to modulate the function of ion channels and the reduced expression of PKC α, ε, and θ protein isoforms observed in hindlimb suspended rats is suggested to contribute to the slow-to-fast transition [29]. Unexpectedly, after prolonged exposure to microgravity, an overall up-regulation of PKC genes was observed in soleus muscle, and partly in EDL, suggesting possible differences between simulated and actual microgravity, rat and mouse, short- and long-term exposure, or a compensatory mechanism of gene expression increase due to the reduced protein kinase C content. Further experiments are needed to verify the various hypotheses.

### Comparison with other missions

The levels of atrophy and phenotype shift observed in mouse soleus after 91 days of space journey appear quite similar to those observed in rodents after short-term mission, suggesting that an equilibrium may be reached already after two weeks of spaceflight. We, however, cannot exclude the possibility that the underlying mechanisms are exactly the same. For instance, we observed no change in atrogin-1 expression after 91-day spaceflight, whereas an up-regulation was observed in mouse soleus after 12 days of spaceflight [13], suggesting a transient activation of the ubiquitin-ligase. Following the same reasoning, we found no change in the expression of two autophagy-related genes after 3 months spaceflight, although recent data demonstrated that the autophagy-lysosome and ubiquitin-proteasome systems may be coordinately regulated during muscle wasting [63], [64]. Yet, autophagy has not been evaluated after short-term spaceflight, and it remains the possibility that autophagy may contribute to the early phases of muscle atrophy, as it does in other muscle disuse conditions [65]. An additional issue that needs further attention is to determine whether recovery from short- and long-term space journeys requires the same time duration, since significant differences were found in recovery periods after short- and long-term hindlimb unloading of rats [30].

In humans, the prolonged exposure to microgravity for up to 6 months causes a 20% reduction of soleus muscle mass [66], largely less than that observed in rodents (35–40%), possibly because of the exercise countermeasures adopted by astronauts. However, the fairly limited muscle atrophy compared to rodents was associated to a substantial drop of muscle force not expected considering the continuous exercise, suggesting that high-intensity exercise programs [66] and possibly pharmaceutical treatments directed to selected molecular targets are needed.

In conclusion, the present study demonstrates a long-lasting sensitivity to microgravity of antigravity soleus muscle throughout the 91 days space mission, while evidencing possible resistance mechanisms to microgravity-induced atrophy of the fast-twitch EDL muscle. Moreover, the results also indicated that IGF-1, NOS1 and IL-6 might represent molecular targets for the development of countermeasures to prevent muscle wasting conditions on the Earth.

## Supporting Information

Figure S1 **Hematoxylin-eosin staining of muscles from mice flown on board ISS and ground-based controls.** Extensor digitorum longus (EDL) and soleus muscle cryosections were stained with hematoxylin-eosin, as indicated in Materials and Methods. No pathological signs, edema, damaged fibers, central nuclei, etc., were evident in all muscles. LAB, mice housed on ground for 91 days in normal laboratory cages; Ground, mice housed on ground for 91 days in MDS (mice drawer system) payload; Flight, spaceflight mice housed in MDS payload for 91 days on board the International Space Station; Cytoplasm inhomogeneity of some samples is attributed to freezing artifacts.
(PDF)

Figure S2 **Myonuclear number in mice flown on board ISS and ground-based controls.** Myonuclei were counted in EDL and soleus muscle cryosections stained with anti-laminin antibodies and DAPI, as described in Materials and Methods. To obtain the actual myonuclei number, the number of satellite cells, identified by Pax7 staining, was subtracted from that of DAPI-positive nuclei inside the laminin staining. Flown soleus muscles show a slightly reduced myonuclear number compared to ground controls. L, mice housed on ground for 91 days in normal laboratory cages (open bars, n = 3, data are expressed as means ± SEM); G, mouse housed on ground for 91 days in the MDS payload (light blue bars); F, spaceflight mouse housed in the MDS payload for 91 days on board ISS (blue bars).
(PDF)

Figure S3 **Laminin staining of muscle fibers of mice flown on board ISS and ground-based controls.** EDL and soleus muscle cryosections were probed with antibodies specific for laminin, as described in Methods. The area inside the laminin staining was utilized to measure muscle fiber CSA. Flown soleus muscle clearly shows a reduced mean cross-sectional area (CSA) compared to on ground controls. L, mice housed on ground for 91 days in normal laboratory cages; G, mouse housed on ground for 91 days in the MDS payload; F, spaceflight mouse housed in the MDS payload for 91 days on board ISS.
(PDF)

Figure S4 **Mean fiber CSA and BW ratio in mice flown on board ISS and ground-based controls.** Ratio between the cross sectional area (CSA) of muscle fibers from EDL and soleus muscles with the body weight (BW) of mice flown for 91 days on board ISS. L, mice housed on ground for 91 days in normal laboratory cages (open bars, n = 3, data are expressed as means ± SEM); G, mouse housed on ground for 91 days in the MDS payload (light blue bars); F, spaceflight mouse housed in the MDS payload for 91 days on board ISS (blue bars).
(PDF)

Table S1 **Quantitative real-time PCR primers and conditions.** RT-PCR primers and techniques utilized to quantitate the expression of the indicated genes. Transcript levels of genes were quantitated either by SYBR Green or Taq polymerase method. HK genes, housekeeping genes; bp, expected product size; T°, annealing temperature.
(PDF)

## References

[1] Schiaffino S, Sandri M, Murgia M (2007). Activity-dependent signaling pathways controlling muscle diversity and plasticity.. Physiology (Bethesda).

[2] Fitts RH, Riley DR, Widrick JJ (2001). Functional and structural adaptations of skeletal muscle to microgravity.. J Exp Biol.

[3] Harrison BC, Allen DL, Girten B, Stodieck LS, Kostenuik PJ (2003). Skeletal muscle adaptations to microgravity exposure in the mouse.. J Appl Physiol.

[4] Jiang B, Ohira Y, Roy RR, Nguyen Q, Ilyina-Kakueva EI (1992). Adaptation of fibers in fast-twitch muscles of rats to spaceflight and hindlimb suspension.. J Appl Physiol.

[5] Ohira Y, Jiang B, Roy RR, Oganov V, Ilyina-Kakueva E (1992). Rat soleus muscle fiber responses to 14 days of spaceflight and hindlimb suspension.. J Appl Physiol.

[6] Ohira Y, Yoshinaga T, Nomura T, Kawano F, Ishihara A (2002). Gravitational unloading effects on muscle fiber size, phenotype and myonuclear number.. Adv Space Res.

[7] Desplanches D, Mayet MH, Ilyina-Kakueva EI, Sempore B, Flandrois R (1990). Skeletal muscle adaptation in rats flown on Cosmos 1667.. J Appl Physiol.

[8] Edgerton VR, Zhou M-Y, Ohira Y, Klitgaard H, Jiang B (1995). Human fiber size and enzymatic properties after 5 and 11 days of spaceflight.. J Appl Physiol.

[9] Ohira Y, Yoshinaga T, Ohara M, Nonaka I, Yoshioka T (1999). Myonuclear domain and myosin phenotype in human soleus following bed rest with or without loading.. J Appl Physiol.

[10] Yamashita-Goto K, Okuyama R, Kawasaki K, Fujita K, Yamada T (2001). Maximal and submaximal forces of slow fibers in human soleus after bed rest.. J Appl Physiol.

[11] Morey-Holton ER, Hill EL, Souza KA (2007). Animals and spaceflight: from survival to understanding.. J Muscoloskelet Neuronal Interact.

[12] Staron RS, Kraemer WJ, Hikida RS, Reed DW, Murray JD (1998). Comparison of soleus muscles from rats exposed to microgravity for 10 versus 14 days.. Histochem Cell Biol.

[13] Allen DL, Bandstra ER, Harrison BC, Thorng S, Stodieck LS (2009). Effects of spaceflight on murine skeletal muscle gene expression.. J Appl Physiol.

[14] Nikawa T, Ishidoh K, Hirasaka K, Ishihara I, Ikemoto M (2004). Skeletal muscle gene expression in space-flown rats.. FASEB J.

[15] Cancedda R, Pignataro S, Alberici G, Tenconi C (2002). Mice Drawer System: phase c/d development and perspective.. J Gravit Physiol.

[16] Masuda H, Tsujimura A, Yoshioka M, Arai Y, Kuboki Y (1997). Bone mass loss due to estrogen deficiency is compensated in transgenic mice overexpressing human osteoblast stimulating factor-1.. Biochem Biophys Res Commun.

[17] Rudnick J, Püttmann B, Tesch PA, Alkner B, Schoser BG (2004). Differential expression of nitric oxide synthases (NOS 1-3) in human skeletal muscle following exercise countermeasure during 12 weeks of bed rest.. FASEB J.

[18] Salanova M, Schiffl G, Blottner D (2009). Atypical fast SERCA1a protein expression in slow myofibers and differential S-nitrosylation prevented by exercise during long term bed rest.. Histochem Cell Biol.

[19] Schiaffino S, Gorza L, Sartore S, Saggin L, Ausoni S (1989). Three myosin heavy chain isoforms in type 2 skeletal muscle fibres.. J Muscle Res Cell Motil.

[20] Danieli-Betto D, Esposito A, Germinario E, Sandonà D, Martinello T (2005). Deficiency of α-sarcoglycan differently affects fast- and slow-twitch skeletal muscles.. Am J Physiol Regul Integr Comp Physiol.

[21] Desaphy J-F, Pierno S, Liantonio A, Giannuzzi V, Digennaro C (2010). Antioxidant treatment of hindlimb-unloaded mouse counteracts fiber type transition but not atrophy of disused muscles.. Pharmacol Res.

[22] Kallen RG, Sheng Z-H, Yang J, Chen LQ, Rogart RB (1990). Primary structure and expression of a sodium channel characteristic of denervated and immature rat skeletal muscle.. Neuron.

[23] Kawano F, Matsuoka Y, Oke Y, Higo Y, Terada M (2007). Role(s) of nucleoli and phosphorylation of ribosomal protein S6 and/or HSP27 in the regulation of muscle mass.. Am J Physiol.

[24] Ohira T, Wang XD, Terada M, Kawano F, Nakai N (2011). Region-specific responses of adductor longus muscle to gravitational load-dependent activity in Wistar Hannover rats.. PLoS ONE.

[25] Wang XD, Kawano F, Matsuoka Y, Fukunaga K, Terada M (2006). Mechanical load-dependent regulation of satellite cell and fiber size in rat soleus muscle.. Am J Physiol Cell Physiol.

[26] Brenman JE, Chao DS, Xia H, Aldape K, Bredt DS (1995). Nitric oxide synthase complexed with dystrophin and absent from skeletal muscle sarcolemma in Duchenne muscular dystrophy.. Cell.

[27] Kobzik L, Reid MB, Bredt DS, Stamler JS (1994). Nitric oxide in skeletal muscle.. Nature.

[28] Ishihara A, Fujino H, Nagatomo F, Takeda I, Ohira Y (2008). Gene expression levels of heat shock proteins in the soleus and plantaris muscles of rats after hindlimb suspension or spaceflight.. J Physiol Sci.

[29] Pierno S, Desaphy JF, Liantonio A, De Luca A, Zarrilli A (2007). Disuse of rat muscle in vivo reduces protein kinase C activity controlling the sarcolemma chloride conductance.. J Physiol.

[30] Desaphy JF, Pierno S, Liantonio A, De Luca A, Didonna MP (2005). Recovery of the soleus muscle after short- and long-term disuse induced by hindlimb unloading: effects on the electrical properties and myosin heavy chain profile.. Neurobiol Dis.

[31] Pierno S, Desaphy JF, Liantonio A, De Bellis M, Bianco G (2002). Change of chloride ion channel conductance is an early event of slow-to-fast fibre type transition during unloading-induced muscle disuse.. Brain.

[32] Musarò A, Dobrowolny G, Rosenthal N (2007). The neuroprotective effects of a locally acting IGF-1 isoform.. Exp Gerontol.

[33] Scicchitano BM, Rizzuto E, Musarò A (2009). Counteracting muscle wasting in aging and neuromuscular diseases: the critical role of IGF-1.. Aging (Albany NY).

[34] Serrano AL, Baeza-Raja B, Perdiguero E, Jardí M, Muñoz-Cánoves P (2008). Interleukin-6 is an essential regulator of satellite cell-mediated skeletal muscle hypertrophy.. Cell Metab.

[35] Washington TA, White JP, Davis JM, Wilson LB, Lowe LL (2011). Skeletal muscle mass recovery from atrophy in IL-6 knockout mice.. Acta Physiol (Oxf).

[36] Naito H, Powers SK, Demirel HA, Sugiura T, Dodd SL (2000). Heat stress attenuates skeletal muscle atrophy in hindlimb-unweighted rats.. J Appl Physiol.

[37] Takeda I, Fujino H, Murakami S, Kondo H, Nagatomo F (2009). Thermal reconditioning prevents fiber type transformation of the unloading induced-atrophied muscle in rats.. J Muscle Res Cell Motil.

[38] Goto K, Honda M, Kobayashi T, Uehara K, Kojima A (2004). Heat stress facilitates the recovery of atrophied soleus muscle in rat.. Jpn J Physiol.

[39] Goto K, Okuyama R, Sugiyama H, Honda M, Kobayashi T (2003). Effects of heat stress and mechanical stretch on protein expression in cultured skeletal muscle cells.. Pflügers Arch.

[40] Ohno Y, Yamada S, Sugiura T, Ohira Y, Yoshioka T (2011). Possible role of NF-κB signals in heat stress-associated increase in protein content of cultured C_2_C_12_ cells.. Cells Tissues Organs.

[41] Kobayashi T, Goto K, Kojima A, Akema T, Uehara K (2005). Possible role of calcineurin in heating-related increase of rat muscle mass.. Biochem Biophys Res Commun.

[42] Miura H, Hashida K, Sudo H, Awa Y, Takarada-Iemata M (2010). Deletion of Herp facilitates degradation of cytosolic proteins.. Genes Cells.

[43] Carlson CJ, Booth FW, Gordon SE (1999). Skeletal muscle myostatin mRNA expression is fiber-type specific and increases during hindlimb unloading.. Am J Physiol.

[44] Haida P, Fowler WM, Abresch RT, Larson DB, Sharman RB (1989). Effect of hindlimb suspension on young and adult skeletal muscle. I. Normal mice.. Exp Neurol.

[45] Criswell DS, Booth FW, DeMayo F, Schwartz RJ (1998). Overexpression of IGF-1 in skeletal muscle of transgenic mice does not prevent unloading-induced atrophy.. Am J Physiol.

[46] Stelzer JE, Widrick JJ (2003). Effects of hindlimb suspension on the functional properties of slow and fast soleus fibers from three strains of mice.. J Appl Physiol.

[47] Caiozzo VJ, Baker MJ, Herrick RE, Tao M, Baldwin KM (1994). Effect of spaceflight on skeletal muscle: mechanical properties and myosin isoform content of a slow muscle.. J Appl Physiol.

[48] Allen DL, Yasui W, Tanaka T, Ohira Y, Nagaoka S (1996). Myonuclear number and myosin heavy chain expression in rat soleus single muscle fibers after spaceflight.. J Appl Physiol.

[49] Hikida RS, Van Nostran S, Murray JD, Staron RS, Gordon SE (1997). Myonuclear loss in atrophied soleus muscle fibers.. Anat Rec.

[50] Kraemer WJ, Staron RS, Gordon SE, Volek JS, Koziris LP (2000). The effects of 10 days of spaceflight on the shuttle Endeavor on predominantly fast-twitch muscles in the rat.. Histochem Cell Biol.

[51] Desplanches D, Mayet MH, Ilyina-Kakueva EI, Frutose J, Flandrois R (1991). Structural and metabolic properties of rat exposed to weightlessness aboard Cosmos 1887.. Eur J Appl Physiol.

[52] Schuenke MD, Reed DW, Kraemer WJ, Staron RS, Volek JS (2009). Effects of 14 days of microgravity on fast hindlimb and diaphragm muscles of the rat.. Eur J Appl Physiol.

[53] Riley DA, Ellis S, Slocum GR, Sedlak FR, Bain JLW (1996). In-flight and postflight changes in skeletal muscles of SLS-1 and SLS-2 spaceflown rats.. J Appl Physiol.

[54] Hansen G, Martinuk KJB, Bell GJ, MacLean IM, Martin T (2004). Effects of spaceflight on myosin heavy-chain content, fibre morphology and succinate dehydrogenase activity in rat diaphragm.. Pflugers Arch - Eur J Physiol.

[55] Stevens L, Firinga C, Gohlsch B, Bastide B, Mounier Y (2000). Effects of unweighting and clenbuterol on myosin light and heavy chains in fast and slow muscles of rat.. Am J Physiol.

[56] Kawano F, Takeno Y, Nakai N, Higo Y, Terada M (2008). Essential role of satellite cells in the growth of rat soleus muscle fibers.. Am J Physiol.

[57] Nakane M, Schmidt HH, Pollock JS, Förstermann U, Murad F (1993). Cloned human brain nitric oxide synthase is highly expressed in skeletal muscle.. FEBS Lett.

[58] Silvagno F, Xia H, Bredt DS (1996). Neuronal nitric oxide synthase-μ, an alternatively spliced isoform expressed in differentiated skeletal muscle.. J Biol Chem.

[59] Suzuki N, Motohashi N, Uezumi A, Fukada S, Yoshimura T (2007). NO production results in suspension-induced muscle atrophy through dislocation of neuronal NOS.. J Clin Invest.

[60] Tidball JG, Lavergne E, Lau KS, Spencer MJ, Stull JT (1998). Mechanical loading regulates NOS expression and activity in developing and adult skeletal muscle.. Am J Physiol.

[61] Yu Z, Zhang P, Hannink M, Stamler JS, Yan Z (2008). Fiber type specific nitric oxide synthase protects against myofibers against cachectic stimuli.. PLoS ONE.

[62] Martin TP, Edgerton VR, Grindeland RE (1988). Influence of spaceflight on rat skeletal muscle.. J Appl Physiol.

[63] Mammucari C, Milan G, Romanello V, Masiero E, Rudolf R (2007). FoxO3 controls autophagy in skeletal muscle in vivo.. Cell Metab.

[64] Zhao J, Brault JJ, Schild A, Cao P, Sandri M (2007). FoxO3 coordinately activates protein degradation by the autophagic/lysosomal and proteasomal pathways in atrophying muscle cells.. Cell Metab.

[65] Masiero E, Agatea L, Mammucari C, Blaauw B, Loro E (2009). Autophagy is required to maintain muscle mass.. Cell Metab.

[66] Fitts RH, Trappe SW, Costill DL, Gallagher PM (2010). Prolonged space flight-induced alterations in the structure and function of human skeletal muscle fibres.. J Physiol.

