# Supplementary figures and images for: Adaptation of Mouse Skeletal Muscle to Long-Term Microgravity in the MDS Mission
Source: PLoS One. 2012 Mar 28;7(3):e33232. doi: 10.1371/journal.pone.0033232 (PMC3314659; doi:10.1371/journal.pone.0033232)

EDL

Soleus

LAB

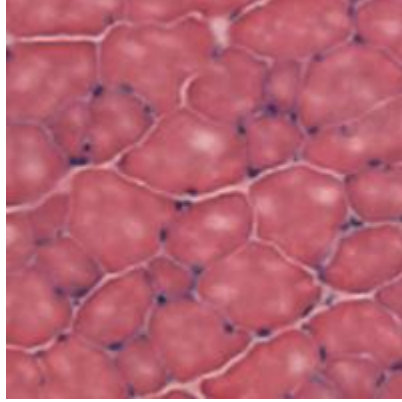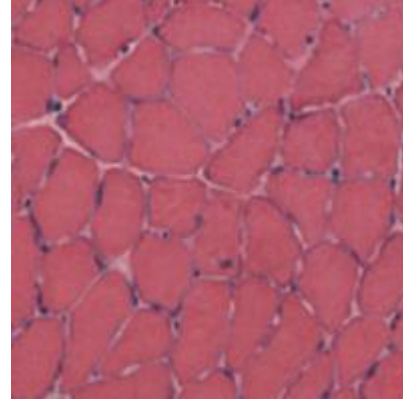

Ground

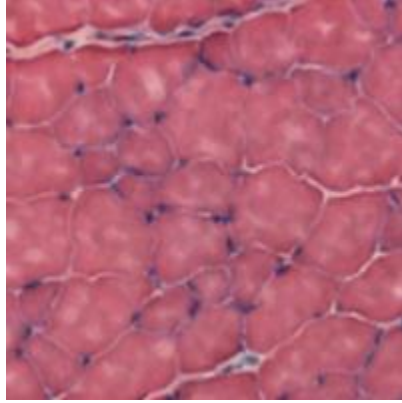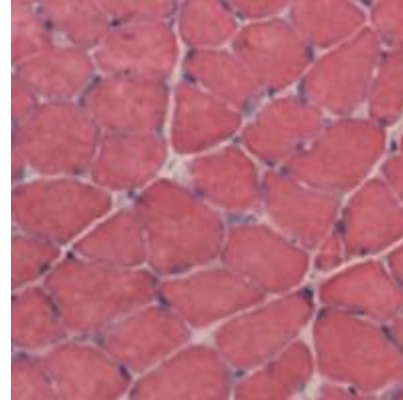

Flight

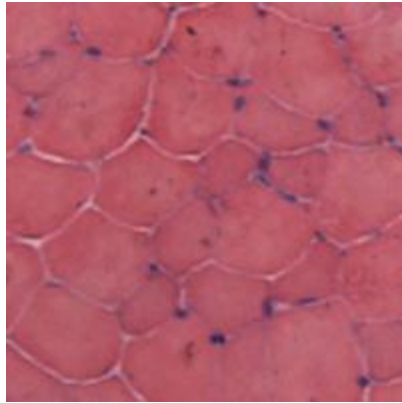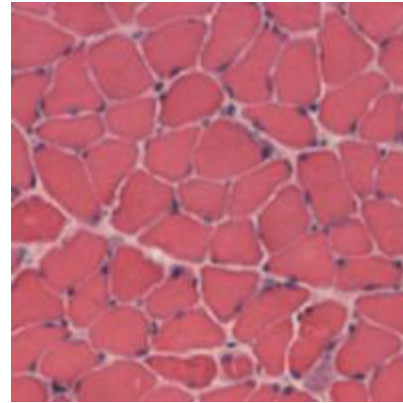

Supplement: Figure S1 — Hematoxylin-eosin staining of muscles from mice flown on board ISS and ground-based controls. Extensor digitorum longus (EDL) and soleus muscle cryosections were stained with hematoxylin-eosin, as indicated in Materials and Methods. No pathological signs, edema, damaged fibers, central nuclei, etc., were evident in all muscles. LAB, mice housed on ground for 91 days in normal laboratory cages; Ground, mice housed on ground for 91 days in MDS (mice drawer system) payload; Flight, spaceflight mice housed in MDS payload for 91 days on board the International Space Station; Cytoplasm inhomogeneity of some samples is attributed to freezing artifacts. (PDF) [file pone.0033232.s001.pdf]

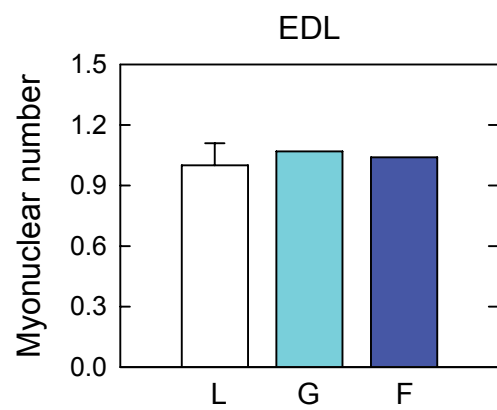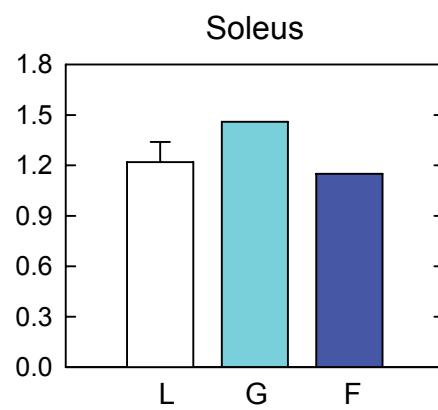

Supplement: Figure S2 — Myonuclear number in mice flown on board ISS and ground-based controls. Myonuclei were counted in EDL and soleus muscle cryosections stained with anti-laminin antibodies and DAPI, as described in Materials and Methods. To obtain the actual myonuclei number, the number of satellite cells, identified by Pax7 staining, was subtracted from that of DAPI-positive nuclei inside the laminin staining. Flown soleus muscles show a slightly reduced myonuclear number compared to ground controls. L, mice housed on ground for 91 days in normal laboratory cages (open bars, n = 3, data are expressed as means ± SEM); G, mouse housed on ground for 91 days in the MDS payload (light blue bars); F, spaceflight mouse housed in the MDS payload for 91 days on board ISS (blue bars). (PDF) [file pone.0033232.s002.pdf]

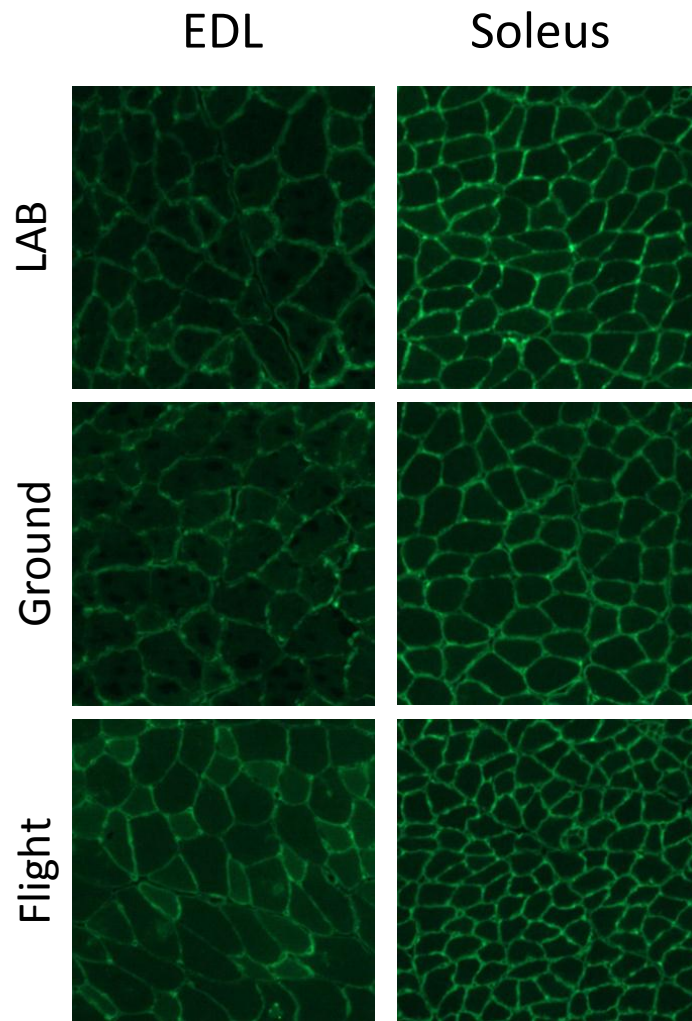

Supplement: Figure S3 — Laminin staining of muscle fibers of mice flown on board ISS and ground-based controls. EDL and soleus muscle cryosections were probed with antibodies specific for laminin, as described in Methods. The area inside the laminin staining was utilized to measure muscle fiber CSA. Flown soleus muscle clearly shows a reduced mean cross-sectional area (CSA) compared to on ground controls. L, mice housed on ground for 91 days in normal laboratory cages; G, mouse housed on ground for 91 days in the MDS payload; F, spaceflight mouse housed in the MDS payload for 91 days on board ISS. (PDF) [file pone.0033232.s003.pdf]

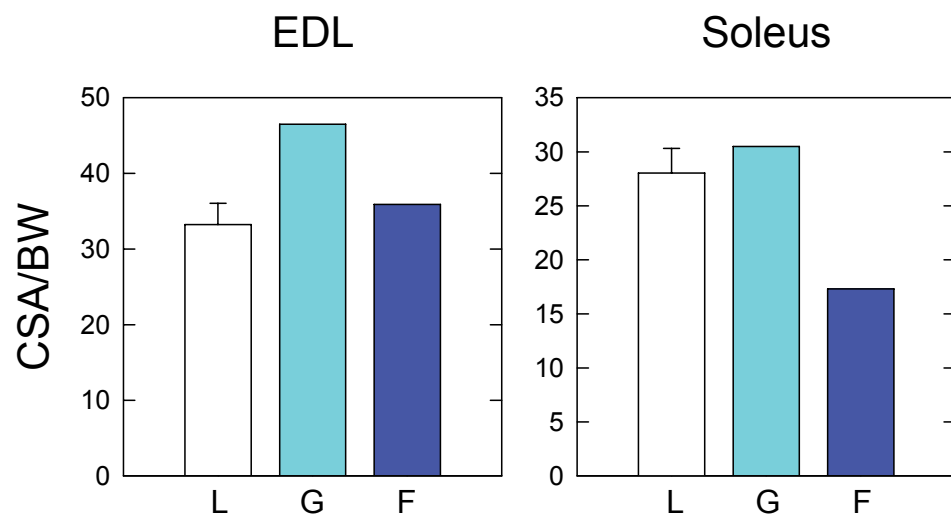

Supplement: Figure S4 — Mean fiber CSA and BW ratio in mice flown on board ISS and ground-based controls. Ratio between the cross sectional area (CSA) of muscle fibers from EDL and soleus muscles with the body weight (BW) of mice flown for 91 days on board ISS. L, mice housed on ground for 91 days in normal laboratory cages (open bars, n = 3, data are expressed as means ± SEM); G, mouse housed on ground for 91 days in the MDS payload (light blue bars); F, spaceflight mouse housed in the MDS payload for 91 days on board ISS (blue bars). (PDF) [file pone.0033232.s004.pdf]
