# Supplementary material for: Adaptation of Mouse Skeletal Muscle to Long-Term Microgravity in the MDS Mission
Source: PLoS One. 2012 Mar 28;7(3):e33232. doi: 10.1371/journal.pone.0033232 (PMC3314659; doi:10.1371/journal.pone.0033232)
Supplement: Table S1 — Quantitative real-time PCR primers and conditions. RT-PCR primers and techniques utilized to quantitate the expression of the indicated genes. Transcript levels of genes were quantitated either by SYBR Green or Taq polymerase method. HK genes, housekeeping genes; bp, expected product size; T°, annealing temperature. (PDF) [file pone.0033232.s005.pdf]

TABLE S1 - Quantitative real-time PCR primers and conditions.

Protein [a]	Genes	Sequence NCBI	Probe Taqman	Forward primer (5' → 3')	Reverse primer (3' → 5')	bp	T°
**HK GENES**							
β-actin	*Actb*	NM_007393.3	NA	CAAACATCCCCCAAAGTTCTAC	TGAGGGACTTCCTGTAACCACT	135	57.7
cyclophilin A	*Ppia*	NC_000077.5	NA	AGCATGTGGTCTTTGGGAAGGTG	CTTCTTGCTGGTCTTGCCATTCC	92	57.7
GAPDH	*Gapdh*	NM_008084.2	NA	TGTGTCCGTCGTGGATCTGA	TTGCTGTTGAAGTCGCAGGAG	150	55.3
HGPT	*Hprt*	NM_013556.2	Mm_0044698_m1 [b]	Unknown	Unknown	65	NA
**SPECIFIC GENES**							
Murf-1 E3 ligase	*Trim63*	NM_001039048.2	NA	ACCTGCTGGTGGAAAACATC	CTTCGTGTTCCTTGCACATC	96	55
Atrogin-1 E3 ligase	*Fbxo32*	NM_026346.2	NA	GCAAACACTGCCACATTCTCTC	CTTGAGGGGAAAGTGAGACG	93	55
LC3β	*Map1lc3b*	NM_026160.4	NA	CACTGCTCTGTCTTGTGTAGGTTG	TCGTTGTGCCTTTATTAGTGCATC	171	57.7
Cathepsin L	*Ctsl1*	NM_001912	NA	GTGGACTGTTCTCACGTCCAAG	TCCGTCCTTCGCTTCATAGG	128	57.7
PGC1-α	*Ppargc1a*	NM_013261.3	NA	GGAATGCACCGTAAATCTGC	TTCTCAAGAGCAGCGAAAGC	88	55
Osgin 2	*Osgin2*	NM_145950.3	NA	CCTTACTGCTCGAGATGGTCCAA	AAACGTCAGGGACTACACGCATAAC	122	47.5
Stress-associated ER protein	*Serp1*	NM_030685.3	NA	TGCCTTGTCGAAACATGTGGA	GACCTAGAACCCTAAGGGCATTTCA	124	39.5
HERP	*Herpud1*	NM_022331.1	NA	TCAAGTGATGGTTTACGGCAAAGA	CACCCATACGTTGTGTAGCCAGAG	140	54.3
PERK	*Eif2ak3*	NM_010121.2	NA	TATGCTACGCACACGGGACAA	ACTCGTTCCATCTGGGTGCTG	152	46.7
Bip (GRP78)	*Hspa5*	NM_022310.2	NA	CTCCACGGCTTCCGATAATCA	TCCAGTCAGATCAAATGTACCCAGA	106	44.3
HSP72	*Hspa1a*	NM_010479.2	NA	CAAGAACGCGCTCGAATCCTA	TCCTGGCACTTGTCCAGCAC	114	57
HSP25	*Hspb1*	NM_013560.2	NA	TCCCTGGACGTCAACCACTTC	AGAGATGTAGCCATGTTCGTCCTG	114	56.1
Pax-7	*Pax7*	NM_011039.2	NA	ACTTCAAGCAGGTGACATCCACA	ACCCTCACGGGCAGATCATTA	93	54.8
NF-κB	*Nfkb1*	NM_008689.2	NA	TGCATATAGCGGCCGGAAG	CCCAAGAGTCGTCCAGGTCATAG	114	57.9
Interleukin 6	*IL6*	NM_031168.1	NA	CCACTTCACAAGTCGGAGGCTTA	GCAAGTGCATCATCGTTGTTCATAC	112	42
Chloride channel 1	*Clcn1*	NM_013491.2	ATTGGCTGAGACACTTGT	TCATGCTCGGTGTCCGAAA	CAGGCGGTGCTTAGCAAGA	192	NA
Sodium channel type IV	*Scn4a*	NM_133199.2	Mm_01258366_m1 [b]	Unknown	Unknown	88	NA
Sodium channel type V	*Scn5a*	NM_021544.3	Mm_01342505_m1 [b]	Unknown	Unknown	85	NA
Sodium channel type I β	*Scn1b*	NM_011322.2	Mm_00441210_m1 [b]	Unknown	Unknown	86	NA
Ca^{2+}-activated K^+ channel (BK)	*Kcnma1*	NM_010610	CAGAGTCCTGGTTGTGTTA	GATATCCGCCCAGACACTGACT	AGTATATTACGAGGGCACCAATGC		NA
ATP-sensitive K^+ channel, Kir6.2	*Kcnj11*	NM_010602.2	CTGGCTCCTAGTGACCTG	CTCATCATCTACCACGTCATCGA	GTTTCTACCACGCCTTCCAAGA	116	NA
ATP-sensitive K^+ channel SUR2A	*Abcc9*	NM_021041.2	CATAGCTCATCGGGTTC	CGGATCGCACGGTCGTA	TAACCAGGTCTGCAGTCAGAATG		NA
ATP-sensitive K^+ channel, SUR1	*Abcc8*	NM_011510.3	Mm_01701349_m1 [b]	Unknown	Unknown	82	NA
PK C, α	*Prkca*	NM_011101.3	Mm_00440858_m1 [b]	Unknown	Unknown	75	NA
PKC, δ	*Prkcd*	NM_011101.3	Mm_00440891_m1 [b]	Unknown	Unknown	58	NA
PK C, θ	*Prkcq*	NM_008859.2	Mm_00435796_m1 [b]	Unknown	Unknown	106	NA
PK C, ε	*Prkce*	NM_011104.3	Mm_00440894_m1 [b]	Unknown	Unknown	64	NA
IGF-1 class 1	*Igf1*	NM_001111275.1	Mm_01233960_m1 [b]	Unknown	Unknown	84	NA
IGF-1 class 1	*Igf1*	NM_010512.4	Mm_01233960_m1 [b]	Unknown	Unknown	84	NA
IGF-1 Ea	*Igf1*	NM_0011112751.1	Mm_00710307_m1 [b]	Unknown	Unknown	68	NA
IGF-1 Ea	*Igf1*	NM_001111276.1	Mm_00710307_m1 [b]	Unknown	Unknown	68	NA

[a], see text for full names; [b], reference number from Applied Biosystems; NA, not applicable
